# HIV prevention: mapping Mozambican people’s views on the acceptability of the widow’s sexual cleansing ritual called pita-kufa

**DOI:** 10.1186/s12914-018-0177-z

**Published:** 2018-09-20

**Authors:** Germano Vera Cruz, Aniceto Mateus, Priscilla S. Dlamini

**Affiliations:** 1 Department of Psychology, Faculty of Education, Eduardo Mondlane University, Campus Universitário Principal, Av. Julius Nyerere, N°3453, CP257, Maputo, Mozambique; 2 Department of Public Health, Higher Institute of Health Sciences, Av. Tomas Ndula, N°977, Maputo, Mozambique; 3 Department of General Nursing, University of Swaziland, Kwaluseni, M201 Swaziland

**Keywords:** HIV, Sexual ritual, Widow cleansing, women’s rights

## Abstract

**Background:**

In Mozambique, the widow is traditionally required to undergo a cleansing ritual called *pita-kufa,* which generally involves several sessions of unprotected sexual intercourse with the brother of her deceased husband. This ritual may play a role in the spread of HIV and reveals, to some degree, the subordinate position to which women are subjected in Mozambican society. Thus, this study’s aim was to map Mozambicans’ views on the acceptability of this ritual, given the gender and public health concerns linked to it.

**Methods:**

A total of 359 Mozambicans participated in the study. The data collection instrument consisted of 18 vignettes describing realistic *pita-kufa* situations, varying as a function of three factors: a widow’s willingness or not to perform the ritual, the perceived effectiveness of the ritual, and the risk level of HIV infection linked to the practice. For each *pita-kufa* situation presented in the vignettes, the participants were asked to rate its acceptability on an 11-point scale. In addition, the participants wrote comments giving their general views on the ritual. A cluster analysis using the K-means procedure was applied to the quantitative raw data to capture different perspectives, and the participants’ written comments were subjected to thematic and frequency content analysis.

**Results:**

From the data gathered though the vignettes, three different perspectives were found: *total unacceptability* (55% of the participants), *conditional acceptability* (29% of the participants) and *unconditional acceptability* (16% of the participants). From the data gathered though the participants’ written comments, it emerged that they thought that the practice of this ritual should *evolve* (61%), *stop* (27%) and be *kept as it is* (12%).

**Conclusion:**

According to the main results, it seems that a large majority of study participants think that this ritual is outdated and needs to evolve in order to minimize the risk of HIV transmission and respect women’s rights.

## Background

In a variety of forms, cleansing rituals for widows are practiced in several Eastern and Southern African countries (e.g., Kenya, Tanzania, Malawi, Zambia, Zimbabwe, and Mozambique) [1–3]. Several studies carried out in those African regions suggest that in many cases, this ritual contributes to the spread of the HIV infection [1, 4–8] and that, in general, this type of traditional practice discourages gender equality, leaving women without the power or means to negotiate safer sex [2, 9, 10].

In Mozambique, traditionally, after the death of his/her wife/husband, the wido*w*/widower has to undergo a cleansing or a purification ritual [10–13]. Although the widow/widower cleansing ritual is observed in all parts of Mozambique, it varies from region to region, depending on the gender of the individual who is subjected to it. In the north (Zambezia, Nampula, Cabo Delgado, and Niassa provinces), it is practiced by the use of water and medicinal plants for both the widow and the widower. They use this mixture to cleanse the body. In the south (Maputo, Gaza and Inhambane provinces) and in the center (Sofala, Manica, and Tete provinces) of the country, the cleansing ritual involves sexual (coital) intercourse for the widow, and the widower is generally “washed” with water mixed with medicinal plants [9, 10, 13]. The widow’s sexual cleansing ritual is called *pita-kufa* in the center of the country and *kutchinga* in the south.

According to the traditional explanation of the widow’s sexual cleansing ritual, after the death of her husband, a woman becomes “dirty”, contaminated by the woe involved in the death of her husband, whose spirit inhabits her body. It is believed that this “dirtiness”, and the misfortune that goes with it, can spread among other members of society. Consequently, the widow must be “washed” in order to remove the contagious “dirtiness” and the curse associated with it, as well as to free her from the shadows of her former life partner [10, 11].

This ritual has been practiced since the pre-colonial period and occurs under the guidance of a traditional healer specialized in the subject. In detail, it consists of unprotected sex involving the widow and a man (the purifier), who generally must be a brother of the deceased [13]. In general, sexual relations are maintained for three consecutive days, often two or three times a day. Under the internal logic of this ritual, the cleansing/purification sexual act is not practiced with condoms because it is the “hot” sexual fluids and the “hot frictions” that are assumed to neutralize the impurity involved in the death of the husband [10, 11].

The strict compliance with the rules of this ritual, in terms of the number of days for having sex, the number of times a day, the place where sexual relations take place and other elements of the protocol, ensures its supposed effectiveness. This, in turn, is believed to enable impurity and contagious woe to be driven out of the community and brings safety, well-being and prosperity to its members. Furthermore, in societies where this ritual is practiced, it is believed that failure to perform (or incorrect performance of) this ceremony may result in disgrace of the family and possibly of the entire community. The disgrace is claimed to be manifested through the spread of illness among family members. In extreme situations, it is manifested by the occurrence of constant deaths in the family and within the community [10].

The practice of this ritual in Mozambican societies reveals, to some degree, the relative subaltern position to which women are subjected compared to men [9, 14, 15]. From the gender inequality perspective, the fact that only widows are subject to sexual (coital) cleansing and not widowers (who generally are purified with water mixed with medicinal plants) is often questioned, and it is a matter of concern for those who advocate equal treatment between men and women [9, 16].

Whether the basis of this practice and the cultural tradition that sustains it are questionable or not, in a country ranked as one of the worst in terms of gender inequality and where HIV prevalence is one of the highest in the world, this ritual has been associated with the violation of women’s rights and the spread of HIV and other sexually transmitted infections (STIs) [1, 4, 6, 8, 17].

In the Gender Inequality Index, Mozambique ranks in 180th position out of 188 countries [18]. The HIV prevalence among the 15–49-year-old population is 10.5% [19]. Despite the intensification of prevention campaigns, the number of new infections continues to increase annually in the south and the center of the country, the regions where *pita-kufa* is practiced [17, 20]. The majority of individuals infected by HIV are women, most of whom are infected through sexual intercourse with infected male partners [21–23].

### The present study

The purpose of this study was to map Mozambicans’ views on the acceptability of *pita-kufa*, taking into account factors such as the widow’s willingness or not to perform the ritual, the ritual’s perceived effectiveness (supposed/reported effectiveness according to the family and the community members), and the risk of HIV transmission linked to it. Given the limited financial and material resources we had, this research was conceived as an exploratory project, likely to inform future studies with different designs.

Although the widow’s sexual cleansing ritual has been widely studied from a descriptive anthropological and sociological perspective [9, 10, 13], to our knowledge no quantitative research has been carried out aimed at examining the population’s views regarding its acceptability. One qualitative study with a small sample (67 subjects) was conducted in southern Mozambique and the results showed that the participants differed in their views regarding the acceptability of the ritual: some considered the practice unacceptable because it raises both gender and public health concerns, some considered the ritual acceptable because it has to do with cultural practice and traditional beliefs and values, and others were indifferent [10]. As far as we know, there are also no quantitative studies reporting the level of willingness of the widow to undergo a sexual cleansing ritual. The few qualitative works researching the widow’s willingness to undergo the ritual carried out in Mozambique revealed that the widows were forced to participate in order to avoid the curse and social stigma of rejecting traditional practices, though others declared that they underwent sexual cleansing by their own will because they believed that the ritual would be of benefit to them and their family members [9, 10, 16]. Thus, given the lack of literature on the acceptability of the *pita-kufa* ritual, it was anticipated that the results of this study would contribute to the current knowledge base in the field.

## Methods

### Participants

The study’s targeted population was Mozambican adults (over 18 years old) living in regions where *pita-kufa* is practiced (in the south and in the center of the county) who could read and understand a simple text in Portuguese, the official language of the country. Individuals under 18 years old were not eligible for the study due to the age required for consent. The participants did not receive any payment (as we did not have funds for this purpose). They were randomly recruited by six research assistants (psychology students) in the two Mozambican regions where *pita-kufa* is practiced. A total of 489 participants were contacted. Of all participants contacted, 359 (189 women and 170 men) agreed to participate. The main reason for refusal was lack of time. The participants’ ages ranged from 18 to 66 years old (*M* = 26.52 years, *SD* = 5.12 years). More detailed demographic information is shown in Table 2.

With regard to knowledge of the ritual, 98.3% of the participants declared that they had already heard about this practice; 67% said that the ritual is still practiced in the community to which they belong; and 36.8% affirmed that they knew someone who had undergone this sexual cleansing process.

### Data collection instrument

This study was conducted using vignettes (scenarios) as data collection material, which were contextualized and plausible stories telling a particular case of a widow’s sexual cleansing ritual. The vignettes were followed by a question and a response scale. In this study, scenarios were utilized instead of simple questionnaires, as scenarios have the advantage of reproducing the description of a particular case in the form of a story. Many variants of the vignettes were written to reflect the manipulated variables and their respective categories. In addition, the use of scenarios allowed for a within-subject factorial design study and performance of cluster analysis on the raw data. This was done in order to find the participants’ different perspectives on the acceptability of the widow’s sexual cleansing ritual, which was the aim of the study. The use of scenario methodology to examine people’s positions, views and judgment on socio-cultural issues is advocated by Anderson [24, 25]. This methodology was already successfully applied in different studies carried out across the world [26], and it has been validated [27].

In this study, the data collection material consisted of 18 cards containing a vignette narrating a particular case of a widow who had undergone a sexual cleansing ritual. The scenarios were composed based on a three within-subject factor design (2 × 3 × 3): *the widow’s willingness or not to undergo this ritual* (really wanted to do the ritual or did it against her real will – she felt obligated to conform to the tradition) × *the ritual perceived effectiveness* (low, intermediary, high) (the ritual’s supposed/reported effectiveness, according to the widow’s family and the community members, regarding the extent to which the widow was “cleaned”, the woe related to the death of her husband was expelled, and the members of the community have benefited from well-being and health) × *the risk of HIV infection linked to the ritual* (low, intermediary, high). The dependent variable was the acceptability of the ritual, and the independent variables were the three factors (the widow’s willingness or not to undergo this ritual, the ritual’s perceived effectiveness, and the risk of HIV infection linked to the ritual). Under each vignette were the question and the response scale. The question was: “*To what extent do you think that the ritual which this widow underwent is acceptable*?” The response scale was an 11-point visual analogue scale with a left-hand anchor of “Totally unacceptable” (0) and a right-hand anchor of “Completely acceptable” (10).

An example scenario is provided below: “*Pita-kufa is a ritual that consists of sexual intercourse involving the widow who recently lost her spouse and a man who in principle must be the brother of the deceased. The ritual is supposed to purify or clean the widow, her family and the community from the misfortune linked to her husband’s death. In general, sexual relations are maintained for three consecutive days, often two or three times a day. The ritual occurs under the guidance of a traditional healer specialized in the subject. For instance, after her husband’s death, Laurinda who lives in Manica was subjected to this type of ritual. She did not want to fulfil this ritual, and she did it only to conform to tradition and to satisfy the demands of her family. During the sexual relations she had with her late husband’s brother (or another person designated for the role), neither she nor her sexual partner used condoms, notwithstanding the fact that both are living in an area where the HIV prevalence rate (16.5%) is among the highest in the country. In the months that followed the realization of this ritual, Laurinda, her family, and the members of the community she lived in experienced moments of well-being and good health; they considered that the ritual had been extremely effective in reaching its goals.*” It is to be noted that in this vignette, the high level of effectiveness of the ritual, which is one of the three parameters of the analysis, is defined by the fact that after its realization, Laurinda, her family and the members of the community experienced moments of well-being and good health. It is also defined by the fact that, according to Laurinda’s family and the community members’ own assessments, the ritual had been extremely effective at achieving its goals.

In addition to the data collection instrument described above, on a separate sheet of paper, participants were asked socio-demographic questions about age, sex, educational level, and living areas (urban or rural). Participants were also asked to write a few sentences giving their general thoughts about the *pita-kufa* ritual, and these comments helped researchers to interpret and discuss the research results.

Before the main research began, a pilot study (30 participants) to test the reliability/validity of the data collection instrument was conducted. The data from the pilot study were subjected to a confirmatory factorial analysis. The three factors and their respective levels were established, and this indicated that participant responses were meaningful and therefore that the methodology was reliable/valid.

### Sampling technique and procedure

Participant enrollment took place in the two most populated cities and in the three most populated villages of each of all six administrative provinces where *pita-kufa* is practiced (a total of 12 towns and 18 villages). Specifically, from February to November 2017, during four consecutive weekends, in each city and village, research assistants positioned themselves along the main streets (on crossroads, near markets, and near other busy places) where they had a high likelihood of finding individuals belonging to all social and professional sectors of the Mozambican population. They selected every third person who passed by. They explained the purpose of the study, said to him/her that they were looking for individuals over 18 years old who could read and understand a simple text in Portuguese to participate in a study about the widow’s sexual cleansing ritual. A sample vignette was given to the person to read. Then, the research assistant requested his/her participation. From those interested, he obtained a signed informed consent. For processing, an appointment was set up at the participants’ home or at the local public university (according to the participants’ convenience). This simple random selection of participants was utilized in order to avoid recruitment bias while trying to have a more purposeful sample of the target population at an inexpensive cost [28]. It is important to note that this recruitment technique is used and considered by the Mozambican Institute for National Statistics [28] as one of the most effective sampling procedures to obtain a socio-demographically diversified group of study participants within the local context. Indeed, in Mozambique, one of the poorest countries in the world, few people have personal cars, and in the large majority of cities and villages, there is no public transportation system. Furthermore, the few individuals who have cars do not use them regularly because they cannot always afford to pay the fuel expenses. Because of that, particularly during weekends, people walk to the markets, to visit their friends or extended families, to go to churches or mosques, to work (those who work weekends), to do errands, etc. Also, in almost all Mozambican cities and villages, there are no more than three main streets through which people usually circulate. Given these circumstances, the probability of meeting all categories of the local population in this manner is high. As a matter of fact, using this enrollment technique, it was possible to obtain a sample consisting of all social and professional sectors of the Mozambican population, with the exception of those purposely excluded (illiterate and under 18 years of age). About 58% of the participants chose to process the study requirements in their private homes, and the remaining subjects chose to come to the local university. In order to maintain privacy, each participant was processed individually, in a vacant room at his/her home or at a local university by one of the two researchers responsible for collecting the data. The data collection materials for the study (vignettes) were written in Portuguese, and this was also the language spoken with the research participants.

The completion procedure itself involved two phases, and it was conducted as recommended by Anderson [24, 25]. In the *familiarization phase*, the researcher explained to the participants what was expected of them: that they had to carefully read each vignette and use the response scale to indicate the degree of acceptability of the *pita-kufa* ritual according to their own opinion. Next, a definition of the “ritual perceived effectiveness” and the way its three categories were articulated in the vignettes was given to the participants. Then, each participant was presented with the 18 vignettes in random order. After completing the ratings, the participant was allowed to compare responses and make changes until satisfied with the entire set of ratings. In the “*for real” completion phase*, each participant was presented with 18 vignettes in random order. They were asked to read and provide rating, but this time, they were neither allowed to compare responses nor allowed to go back and make changes. The participants took 45–50 min to complete the ratings.

### Compliance with ethical standards

The research was done according to the requirements stipulated for a study of this kind in Mozambique. An informed consent was signed by each participant, and their anonymity was guaranteed in that the results were reported in aggregate form, without tracing responses back to each individual. Each participant was told that he/she was free to withdraw from the research study at any time, without giving a reason and without prejudice. The Ethics Committee of the Eduardo Mondlane University (Mozambique) approved the study before it was undertaken.

### Statistical analysis

First, each rating made by the participants was converted into a numerical value expressing the distance between the point on the response scale and the left anchor serving as an origin. These numerical values were then subjected to graphical and statistical analyses. A cluster analysis using the K-means procedure recommended by Hofmans and Mullet [29] was applied to the raw data to capture potentially different perspectives. Second, separate analyses of variance (ANOVAs) were conducted on the data of each cluster with widow’s willingness or not to undergo this ritual × the perceived effectiveness of the ritual × the risk of HIV infection linked to the ritual, a 2 × 3 × 3 design. Finally, we performed the *χ*2 test to determine the cross-sectional associations between the demographic characteristics and the participants’ judgments. The sentences written by the participants on a separate sheet of paper were subjected to thematic and frequency content (discourse) analysis.

## Results

**Fig. 1:**
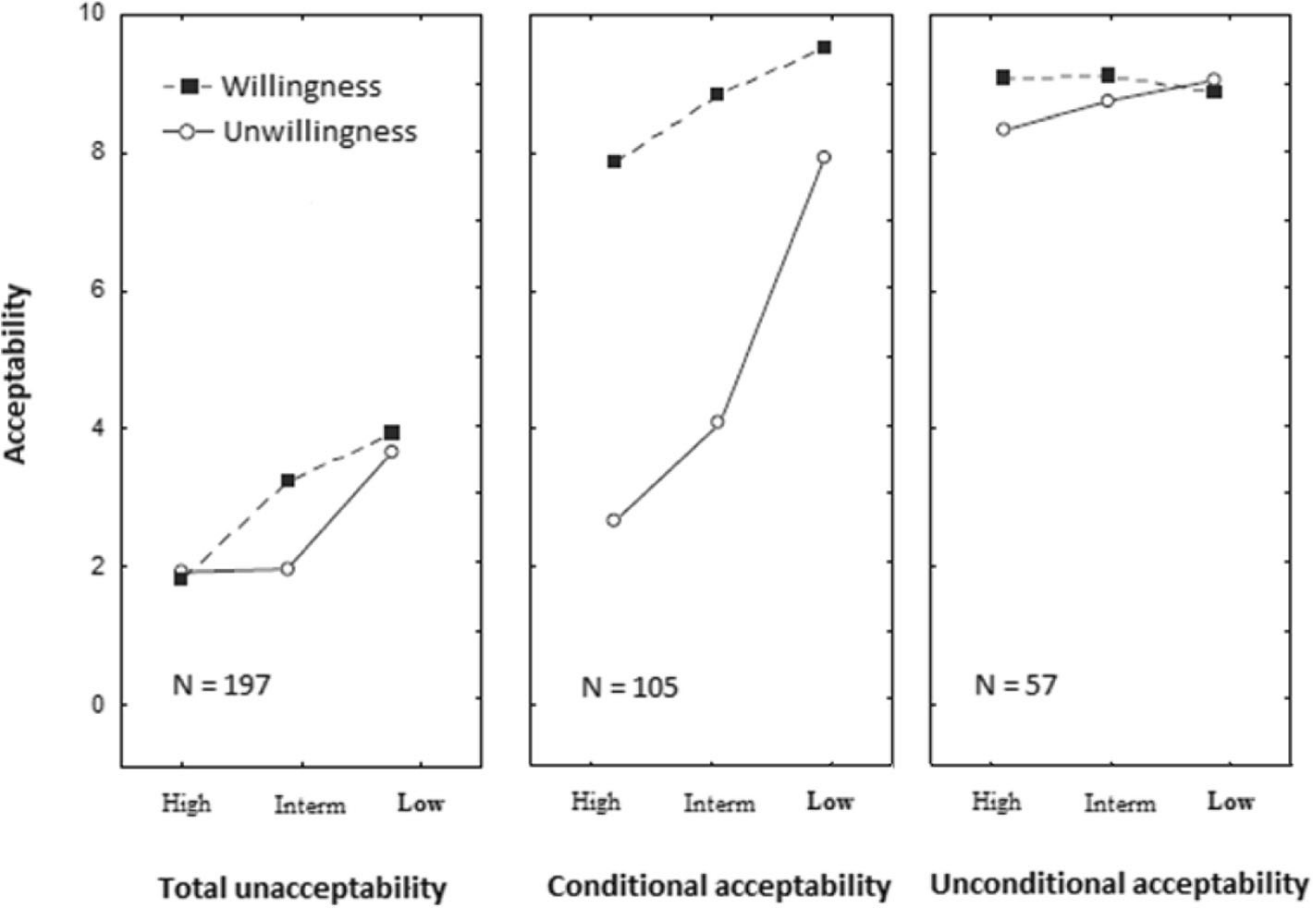
Patterns of Results Corresponding to the Three Clusters. Note, Mean ratings of the widow’s sexual cleansing ritual acceptability as a function of the risk of HIV infection and the widow’s willingness or unwillingness to undergo the ritual. In each of the panel, the acceptability is on the vertical axis, the three levels of the HIV infection risk are on the horizontal axis, and one of the two lines corresponds to the widow’s willingness while the other corresponds to widow’s unwillingness. Each panel corresponds to one cluster: total unacceptability, conditional acceptability, and unconditional acceptability.

A three-cluster solution was retained because it was the one that produced the most meaningful findings. The main patterns of ratings corresponding to these clusters are shown in Fig. 1. The detailed results of the ANOVAs between the three factors and each cluster are shown in Table 1. The associations between the demographic characteristics and the participants’ judgments are presented in Table 2.

**Table 1:** Main Results of the ANOVA Performed at the Cluster Level

	df effect	MS effect	df error	MS error	*F*	*p*	Eta^2^_p_
Cluster total unacceptability							
Widow’s willin (W)	1	117.33	195	2.98	13.59	.067	.00
Ritual perc effecti (E)	2	17.65	390	12.45	4.89	.089	.07
HIV risk (R)	2	112.87	390	32.55	4.12	.060	.22
Custer conditional acceptability							
Widow’s willin (W)	1	324.68	103	10.98	25.00	.001	.34
Ritual perc effecti (E)	2	20.53	206	20.87	0.89	.063	.02
HIV risk (R)	2	32,028.91	206	19.78	144.66	.001	.68
W x R	3	898.07	309	57.87	43.12	.001	.62
Custer unconditional acceptability							
Widow’s willin (W)	1	37.05	55	8.24	3.98	.001	.14
Ritual perc effecti (E)	2	21.42	110	21.54	0.99	.068	.05
HIV risk (R)	2	50.23	110	5.92	6.77	.001	.11

*Willin* willingness, *perc effecti* perceived effectiveness. Significance level = *p* <.05

**Table 2:** Participants’ demographic characteristics and clusters

Characteristics	Clusters			Total
	Total unacceptability	Conditional acceptability	Unconditional acceptability	
Age: X^2^(4) = 10.63, *p* = .031				
18–30 years	79 (50)	58 (37)	20 (13)	157 (100)
31–47 years	70 (63)	22 (20)	19 (17)	111 (100)
48–66 years	48 (52)	25 (27)	18 (21)	91 (100)
Gender: X^2^(2) = 14.35, *p* = .000				
Male	83 (49)	47 (28)	40 (23)	170 (100)
Female	114 (60)	58 (31)	17 (9)	189 (100)
Education: X^2^(2) = 12.50, *p* = .001				
Primary and secondary	98 (48)	63 (31)	43 (21)	204 (100)
Post-secondary and university degree	99 (64)	42 (27)	14 (9)	155 (100)
Living areas: X^2^(2) = 11.0, *p =* .004				
Urban areas	108 (54)	69 (35)	22 (11)	199 (100)
Rural areas	89 (56)	36 (43)	35 (22)	160 (100)
Total	197 (55)	105 (29)	57 (16)	359 (100)

Figures in parentheses are percentages calculated for each row. Significance level = *p* <.05

### Mozambicans’ views on the acceptability of Pita-kufa

For 197 participants (55%), the ratings were always low (*M* = 3.51). None of the three factors (widow’s willingness, perceived effectiveness of the ritual, risk of HIV infection) considered in this study had significant correlation to it (see Table 1). This cluster was called *total unacceptability*.

For 105 participants (29%), the ratings strongly varied as a function of the widow’s own willingness or not to undergo the ritual and the risk of HIV infection linked to the ritual; this cluster was called *conditional acceptability* (*depends on the widow’s willingness*/*risk of HIV infection*). The widow’s willingness × risk of HIV infection interaction was significant (see Table 1). When the risk of HIV infection was high, the ritual unacceptability was always high (mean values ranging from 8.10 to 9.78). When this risk was low, acceptability was strongly associated with the widow’s willingness (mean values ranging from 2.32 to 8.10).

For 57 participants (16%), the ratings were always high (*M* = 8.71). This cluster was called *unconditional acceptability*. Individuals from this cluster strongly endorsed this practice regardless of the widow’s willingness, the perceived effectiveness level of the ritual or the risk of HIV infection.

Finally, it is important to note that the perceived effectiveness of the ritual factor was not significantly related to participants’ judgments of the *pita-kufa*’s acceptability (see Table 1).

### Associations between the participants’ demographic characteristics and their judgments

According to the results of the *χ*2 test, there was a significant correlation between the participants’ age and their judgments – X^2^(4) = 10.63, *p* = .031 – with the younger participants generally judging the ritual as totally unacceptable and the older participants stating that it was conditionally acceptable. Likewise, there was a significant factor related to gender on the participants’ judgments – X^2^(2) = 14.35, *p* = .000 – with the female participants generally judging the ritual as totally unacceptable more than the male participants. The participants’ educational level was also a factor significantly associated with their views concerning the acceptability of the ritual – X^2^(2) = 12.50, *p* = .001 – with those who have post-secondary and university degrees endorsing total unacceptability more than those who have only primary and secondary levels of education. Finally, participants from rural areas tended to significantly adhere to the unconditional acceptability perspective more than those residing in urban areas – X^2^(2) = 11.0, *p* = .004.

### Results of the participants’ written comments on Pita-kufa

A total of 311 participants (out of 359) wrote a few sentences giving their general thoughts about the *pita-kufa* ritual. The thematic and frequency content analysis carried out on these sentences revealed three basic themes.

#### Update the practice (61% of the frequency)

Here are some written comments (translated into English) made by the participants with regard to this theme: “*I think that this ritual is an outdated practice and does not fit well in the present social reality, it might be good to update the way it is practiced*”, wrote a 25-year-old woman; “*I think that it would be better to introduce some changes in the way this ritual is presently practiced so that it does not pose any risk of HIV transmission*”, wrote a 29-year-old women; “*For me, the practice should evolve so that it respects the will of the widow and her human rights*”, wrote a 33-year-old man. This view was equally expressed by female and male participants (51% and 48.5%, respectively).

#### Stopping the practice (27% of the frequency)

Here are some sentences related to this theme: “*We must stop practicing this kind of ritual. While in the past there must have been some justification to do it, the social reality has changed, and we do not need this anymore*”, wrote a 24-year-old man; “*Can someone tell me why men are not subjected to this sexual cleansing ritual in the same ways as women are? Please stop sexually exploiting women’s bodies. We need equality between men and women, not this outdated ritual*”, wrote a 32-year-old woman; “*It is a dangerous practice, since it poses a risk of HIV transmission, and I do not believe in its effectiveness. It is time to stop it*”, wrote a 19-year-old woman. Most of the participants who expressed this view were female (57%).

#### Keep the practice as it is (12% of the frequency)

Here are some sentences related to this theme: “*Even if some people do not understand it, there is a reason this ritual is practiced the way it is, and it is for the good of the community. There is nothing to be changed*”, wrote a 43-year-old man; “*This is part of our traditions and I think we need to keep our traditions alive, as otherwise, everything will fall apart*”, wrote a 37-year-old woman; “*Let us not be influenced by the Westerners. Let us keep our culture, our traditions, and our beliefs as they are*”, wrote a 41-year-old man. Most participants who expressed this view were male (65%).

## Discussion

Overall, the results of this study showed that among participants there were three different perspectives on the acceptability of the ritual: total unacceptability, conditional acceptability, and unconditional acceptability. The qualitative study conducted prior to this study [10] also showed that the participants had different views (total acceptability, complete acceptability, and indifferent) with regard to the ritual. However, due to the methods used, the findings of the present study are much more precise. While suggesting that different perspectives regarding the acceptability of the widow’s sexual cleansing ritual coexist within the target population, the results highlight that those perspectives vary as a function of socio-demographic characteristics.

Specifically, the results of this study showed that (a) the majority of the participants think that this ritual is unacceptable regardless of the three factors considered, (b) the majority of the participants felt that the *pita-kufa* ritual is an outdated practice that does not fit well in present social reality and that changing or updating the practice might be beneficial, (c) a considerable number of participants felt that this practice must be stopped because it undermines women’s rights and it does not represent equality of treatment between women and men. The concern that the *pita-kufa* violates women’s rights is consistent with the result of the previous qualitative research which showed that a significant number of widows who underwent the ritual declared to have done so by force [9, 10, 16]. It is worth noting that most of the participants who expressed the view that the ritual was unacceptable and its practice must stop were women, younger, and with a higher level of education compared to the other participants. Earlier studies [20, 21] concluded that women and young highly educated individuals are most concerned by the significant level of gender inequality and the violation of women’s rights in Mozambican society.

Approximately one third of participants were of the opinion that the ritual is more or less acceptable, provided that the widow really wants to do it, and especially if it poses no risk of HIV infection. The participants who supported this view were seemingly concerned about women’s rights [10] and the problems associated with the gender inequality [18]. They were also concerned by the public health problems (given the high level of HIV prevalence in the country [19]) that may be linked to the practice of this ritual. While suggesting that its practice should be maintained, they also suggested that some changes should be made.

Only 16% of the participants supported the widow’s cleansing ritual as it is currently practiced. The comments written by those participants suggest that they were from a segment of the Mozambican population, evidenced in previous studies [10, 12, 13], who were deeply attached to traditions and a belief system that links the well-being of the family/community to the respect of traditional socio-cultural precepts as well as the accomplishment of rituals that are linked to those precepts.

Several factors related to this study method should be considered when interpreting the findings. First, although the participants of this study were recruited randomly on the streets, the sample was of moderate size and came only from the regions where the ritual is practiced. Moreover, since vignettes were used for data collection, illiterate individuals were effectively excluded. Nevertheless, it is important to stress that all social and professional categories of the Mozambican population were present in the sample. Second, the participants responded to realistic scenarios but not to real situations. The use of vignettes, however, is useful: (a) it makes possible the assessment of immediate reactions to a situation, (b) it standardizes the stories across participants, (c) it permits statistical analyses to examine how people weight and combine separate factors, and (d) it allows the finding and characterization of different patterns of responses in terms of clustering different perspectives.

### Implications

The results of this study can give governmental authorities a basis to engage with community leaders in areas where this ritual is still observed. The results may help community leaders consider the possibility of transforming the way this ritual is traditionally practiced. Changes may include considering the willingness of the stakeholders (including the widows), recognizing the necessity of minimizing the risk of HIV transmission, and making changes or replacing elements of the ritual with other less problematic practices.

## Conclusion

In summary, the participants who think that this ritual is acceptable and who wish to maintain its practice as is are largely in the minority in this study. The results of this study are relevant because they show that the majority of participants feel that this ritual is unacceptable and changes should be introduced in the way it is practiced to address the gender, human rights, and public health concerns linked to it. To our knowledge, in other African countries where the widow’s sexual cleansing ritual is practiced, no prior study has quantified these perspectives. Thus, the results of this study contribute to the literature in the field and create a basis for other studies with different designs. Another main value of this study is the revelation that different perspectives concerning the acceptability of the *pita-kufa* co-exist among participants from the same Mozambican culture and that simple techniques are available for characterizing them precisely.

Future studies should focus on examining the acceptability of the widow’s sexual cleansing ritual, using real-life *pita-kufa* situations and a sample that is more representative of the Mozambican population. In addition, future research must try to examine to what extent the proponents of this ritual (widows, men whose wives are likely to undergo this ritual following their deaths, traditional healers, community leaders, etc.) are willing to introduce changes in the way *pita-kufa* is practiced. Changes in the ritual would address the gender and the public health concerns linked to it, namely, the spread of sexually transmitted infections, gender inequality, and the violation of women’s rights.

## Abbreviations

ANOVA: Analysis of variance
DF: Degree of freedom
HIV: Human immunodeficiency virus
MS: Mean squared
STIs: Sexually transmitted infections
